# Inhibition of Exo-miR-19a-3p derived from cardiomyocytes promotes angiogenesis and improves heart function in mice with myocardial infarction via targeting HIF-1α

**DOI:** 10.18632/aging.103563

**Published:** 2020-12-11

**Authors:** Lianping Gou, Cheng Xue, Xiaoyan Tang, Zhiyuan Fang

**Affiliations:** 1 Department of Cardiovascular Medicine, Affiliated Hospital of North Sichuan Medical College, Nanchong, Sichuan Province, China; 2 Department of General Practitioner, Affiliated Hospital of North Sichuan Medical College, Nanchong, Sichuan Province, China; 3 Department of Medicine, Shanxi Provincial People’s Hospital, Xi’an, Shanxi Province, China

**Keywords:** exosome, myocardial infarction, miR-19a-3p, HIF-1α, angiogenesis

## Abstract

Background: Myocardial infarction (MI), a common presentation for cardiovascular disease, is caused by reduction of blood flow and oxygen supply and is one of the main causes of death worldwide. MicroRNAs participate in multiple physiological and pathological processed and play crucial role in myocardial infarction.

Results: qRT-PCR analysis showed that expression level of miR-19a-3p was increased in serum of patient with MI. *In vitro* study indicated that the miR-19a-3p level was upregulated in response to H_2_O_2_ treatment and transferred by exosome, and then, uptake occurred in endothelial cells. Furthermore, western blot and immunostaining showed that treatment of exosome enriched miR-19a-3p suppressed the proliferation of endothelial cells and induced cell death, which was inhibited by AMO-19 transfection. Administration of antagomiR-19a-3p promoted angiogenesis and improved heart function of MI mice. Moreover, miR-19a-3p overexpression downregulated the protein level of HIF-1α and transfection of si-HIF-1α reversed the promotion of endothelial cells proliferation caused by AMO-19 transfection. In addition, antagomiR-19a-3p treatment accelerated angiogenesis and infection of AAV5-shHIF-1α inhibited that effect in MI mice.

Conclusions: In conclusion, our finding indicated that miR-19a-3p inhibited endothelial cells proliferation and angiogenesis via targeting HIF-1α and attenuated heart function of mice after MI, and suggested a new mechanism of cell-to-cell communication between cardiomyocytes and endothelial cells.

## INTRODUCTION

Acute myocardial infarction is the death of cardiomyocytes resulted from acute and persistent ischemia and hypoxia of coronary artery. It is one of the leading causes of the mortality and morbidity worldwide. Blood flow Acute thrombotic obstruction in coronary arteries was involved in the process of myocardial infarction and results the cardiomyocytes injury, which leads to heart failure and sudden death [1]. Extensive studies have shown that the decrease of cardiac blood perfusion leads to the necrosis and apoptosis of cardiac myocytes, which then activates the proliferation of cardiac fibroblasts, leads to myocardial remodeling and further damage of heart function. Therefore, promoting angiogenesis and increasing blood supply in the infarcted area is very important for promoting the survival of myocardial cells, reducing the infarcted area and improving cardiac function and is promising therapeutic approaches for improving prognosis of patients with myocardial infarction [2].

MicroRNAs (miRNAs) are a kind of noncoding RNA that participates multiple kinds of physiological and pathophysiological process such as apoptosis, proliferation, differentiation and cell cycle and so on. Massive research showed the important effects of miRNAs during the development of cardiovascular diseases. The study of Ivey et al shows that the expression of miR-133 and miR-1 were decreased in the myocardium of mice with constriction of aorta or myocardial specific overexpression of mutant Akt kinase and rats with exercise overload [3]. Porrello ER et al indicates that miR-195 overexpression inhibits neonatal heart regeneration and postnatal inhibition of the miR-15 family induces cardiomyocyte proliferation and improves cardiac function after MI [4]. What’s more, accumulating evidence indicated that miRNAs are also important for regulating the process of myocardial infarction. For example, miR-124 inhibition reduces apoptosis of cardiomyocyte following myocardial infarction by targeting STAT3 [5]. Knockdown of miR-26a promotes autophagy and alleviates cardiac injury in mice with myocardial infarction. In addition, a lot of study reveal the function of miRNAs as a novel kind of mediator of cell communication transferring by extracellular vesicles especially the exosome, which contains complicated RNAs and proteins with a diameter of 30~150 nm. The role of circulating miRNAs is widely reported on regulation of cancer development and miR-19a-3p family is one of the most important one among them. Besides working as a mediator of the angiogenesis and extensive invasion of malignancies, miR-19 was also shown to play important role in heart disease. For example, miR-19 inhibited apoptosis and promoted proliferation of cardiomyocytes. Upregulation of miR-19 reduces the formation of sprout of endothelial cells and regulates the expression level of cyclin D1 and fibroblast growth factor receptor 2 to block the cell cycle [6]. However, the role of miR-19 in cell-cell communication between cardiac and vascular cells and the underlying mechanism is still poorly understood.

In this study we identified the novel role of miR-19a-3p in the crosstalk between cardiomyocytes and endothelial cells via paracrine pathway that regulates angiogenesis by targeting HIF-1α in myocardial infarction mice. This study reveals a novel way for prevention and treatment of myocardial infarction and improvement of heart function.

## RESULTS

### Expression level of miR-19a-3p is upregulated after MI or treatment of H_2_O_2_.

**Figure 1:**
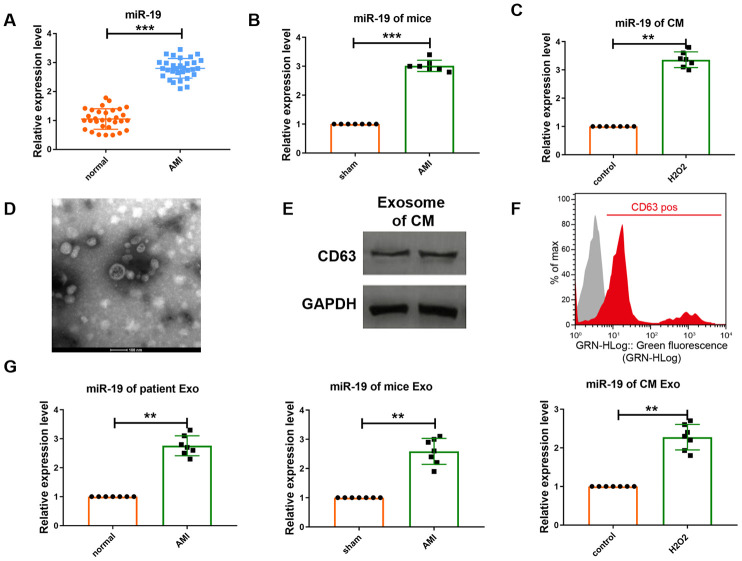
**Expression level of miR-19a-3p is upregulated in response to MI or H_2_O_2._.** (**A**–**C**) qRT-PCR analysis of miR-19a-3p level in patients’ serum, mice serum and culture medium of CM. U6 was used as the control. (**D**) Electron microscopy image of CM–derived exosomes, showing a size of approximately 30 to 150 nm in diameter. Scale bar: 100 nm. (**E**) Western blot analysis of the protein level of CD63. (**F**) Flow cytometry analysis of CD63 of CM-derived exosomes. CM-derived exosomes were immunostained against CD63 (red curve) and compared with the appropriate isotype control (gray curve). (**G**) qRT-PCR analysis of miR-19a-3p level in exosomes derived from patients’ serum, mice serum and culture medium of CM. U6 was used as the control. **P < 0.01 and ***P < 0.001. All experiments were performed more than 3 biological repeats.

Recent study has shown that miRNAs are actively secreted in extracellular vesicles especially exosome, which highlights their underlying effect to act as paracrine signaling molecules [7]. Here we first detected the serum level of miR-19a-3p in patients with acute myocardial infarction (AMI). Real-time PCR (RT-PCR) analysis indicated that the level of miR-19a-3p was increased in patient’s serum compared to normal control (Figure 1A). To further analyze the expression of miR-19a-3p, we then constructed the myocardial infarction (MI) mice model. The result detected by qRT-PCR was the same as before (Figure 1B) Furthermore, we also collected the culture medium of cardiomyocytes and analyzed the level of miR-19a-3p. As a result, it also upregulated in response to the stimulation of H_2_O_2_ (Figure 1C). These results indicate that myocardial infarction was one of the reasons to induce the expression of miR-19a-3p. And then, we want to confirm whether miR-19a-3p was transferred by exosome. Exosomes were separated from culture medium of cardiomyocytes using ultracentrifugation. Electron microscopic analysis showed a typical size of 30-150 nm and a characteristic cup-shaped morphology (Figure 1D), that has been seen as a majority of isolated exosomes [8]. Then, western blot analysis was used to detect the expression of exosome marker CD63 (Figure 1E). Flow cytometry analysis (FACS) also confirmed the expression of CD63 (Figure 1F). Moreover, miR-19a-3p expression was examined from serum of MI patient and mice and culture medium of cardiomyocytes, respectively, and actually increased (Figure 1G). These results indicate that miR-19a-3p secreted form cardiomyocytes were upregulated in response to MI and H_2_O_2_.

### Downregulation of miR-19a-3p promotes endothelial cells survival and proliferation.

**Figure 2:**
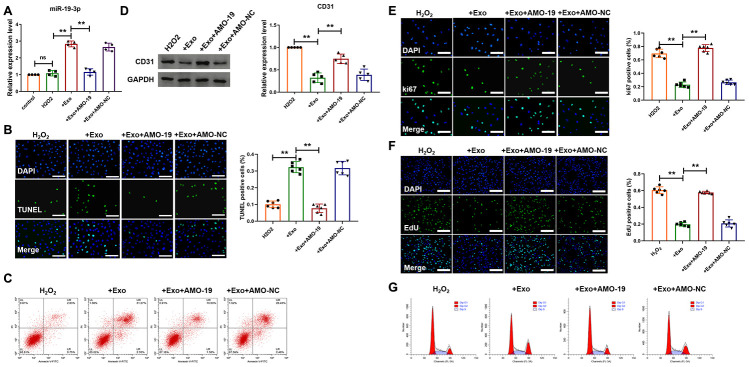
**Downregulation of miR-19a-3p promotes endothelial cells survival and proliferation.** (**A**) qRT-PCR analysis of miR-19a-3p level was measured in endothelial cells. U6 was used as the control. (**B**) TUNEL staining of endothelial cells after treatment of exosomes derived from CM-culture medium. (**C**) Flow cytometry was performed to detect apoptosis of endothelial cells. (**D**) Western blot analysis of expression level of CD31. (**E**) Immunofluorescence staining of ki67 in endothelial cells. (**F**) EdU staining in endothelial cells to show the effect of miR-19a-3p on proliferation of endothelial cells. (**G**) Flow cytometry was performed to detect cell cycle of endothelial cells. Exosome was derived from CM-culture medium. AMO-19 was transfected to inhibit the level of miR-19a-3p. **P < 0.01. All experiments were performed more than 3 biological repeats. Scar bar = 50μm.

Next, we want to determine whether miR-19a-3p from cardiomyocytes could act on endothelial cells by exosome pathway. First, neonatal mice cardiomyocytes were subject to H_2_O_2_ (50 μΜ) for 3 h to simulate ischemic injury of cardiomyocytes *in vitro*. And then, exosomes were separated from condition medium of cardiomyocytes and added to the endothelial cells culture medium for 48 h, in which cells were also treated with H_2_O_2_ for 3 h. AMO-miRNA is a kind of anti-miRNA-oligonucleotides that causes the degradation of target miRNA. AMO-19 was transfected to inhibit the expression of miR-19a-3p. As shown in Figure 2A, there was little difference of miR-19a-3p level between endothelial cells after H_2_O_2_ treatment and control group. However, Addition of exosome increased miR-19a-3p level of endothelial cells and it was decreased after AMO-19 transfection (Figure 2A). These data indicate that exosome containing miR-19a-3p from cardiomyocytes can be uptake by endothelial cells. Then we wanted to explore the function of miR-19a-3p in endothelial cells in ischemic condition *in vitro*. TUNEL staining and flow cytometry was performed to detect endothelial cells apoptosis and addition of exosome elevated H_2_O_2_ induced endothelial cells apoptosis and AMO-19 transfection inhibited endothelial cells apoptosis (Figure 2B and 2C). Furthermore, western blotting showed the downregulation of endothelial cell marker CD31 in exosome addition group. Transfection of AMO-19 elevated the expression level of CD 31 (Figure 2D). Meanwhile, immunofluorescence staining analysis showed that ki-67 positive staining cells’ number were decreased after the treatment of exosome from cardiomyocytes compared to no treatment group. Giving AMO-19 significantly diminished that effect and promote the proliferation of endothelial cells (Figure 2E). In addition, treatment of exosomes collected from cardiomyocytes significantly alleviated cell cycle of endothelial cells and AMO-19 transfection abolished the inhibitory effect (Figure 2F and 2G). These data suggest that inhibition of miR-19a-3p promotes the survival and proliferation of endothelial cells in ischemic injury *in vitro*.

### Inhibition of miR-19a-3p promotes angiogenesis in mice with myocardial infarction.

**Figure 3:**
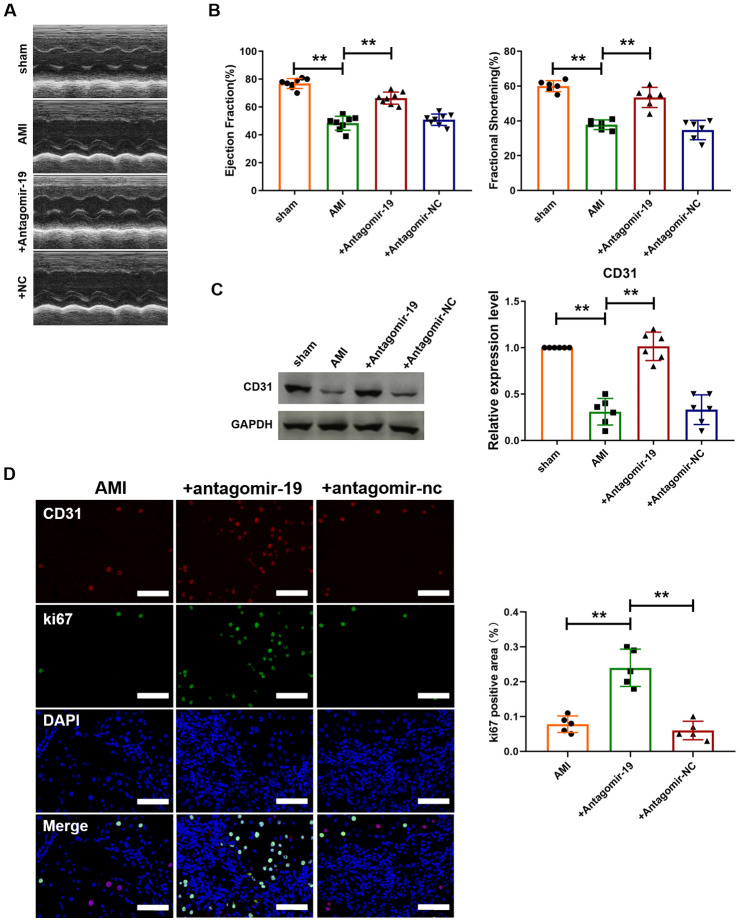
**Inhibition of miR-19a-3p promotes angiogenesis and improves heart function in mice with MI.** (**A**) Representative echocardiographic assessment images of heart function of mice with MI. (**B**) Ejection fractions (EF%) and fractional shortening (%). (**C**) Western blot analysis of expression level of CD31 in left ventricle of mice. (**D**) Immunofluorescence staining of ki67 in peri-infarct area of left ventricle. **P < 0.01. All experiments were performed more than 4 biological repeats. Scar bar = 50μm.

We further investigated the effect of silencing miR-19a-3p on heart function of MI mice. As shown in Figure 3A, echocardiographic assessment was performed to determine the systolic and diastolic function of heart. Administration of antagomiR-19a-3p reversed the damage of heart contractility caused by MI. However, antagomir-NC had no effect. In addition, Fractional Shortening (FS) and Ejection Fraction (EF) were also detected (Figure 3B). And compared with MI group, both of them were recovered after miR-19a-3p silence. In contrast, EF and FS values were not changed in antagomir-NC transfected hearts after MI (Figure 3B). Then, we further examined the effect of miR-19a-3p on angiogenesis in mice MI model. MI significantly decreased the expression level of CD31 in mice heart. In contrast, the expression of CD31 in antagomiR-19a-3p transfected hearts were elevated compared with un-transfected group after MI (Figure 3C). In addition, immunofluorescence staining analysis indicated that the number of ki67 and CD31 double positive staining cells were increased in MI mice with administration of antagomiR-19a-3p compared to no-treat group, which suggested the proliferation of endothelial cells (Figure 3D). Collectively, these data suggest that miR-19a-3p inhibition promotes the proliferation of endothelial cells and angiogenesis and improves heart function.

### Increased expression of HIF-1α is involved in miR-19a-3p downregulation-mediated proliferation of endothelial cells

**Figure 4:**
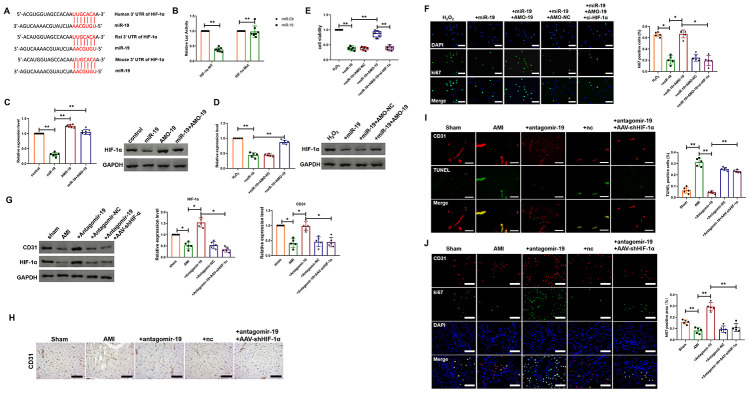
**miR-19a-3p reduces endothelial cells proliferation and attenuates heart function by targeting HIF-1α.** (**A**) The predicted binding site of miR-19a-3p on the 3’ UTR of HIF-1α gene. (**B**) Luciferase reporter activities of chimeric vectors carrying the luciferase gene and a fragment of the 3’ UTR of HIF-1α containing the wild type or mutant miR-19a-3p binding sites. (**C**) Western blot analysis of expression level of HIF-1α of endothelial cells transfected with miR-19a-3p or AMO-19. (**D**) Western blot analysis of expression level of HIF-1α of endothelial cells in response to H_2_O_2_ treatment with or without transfection of miR-19a-3p or AMO-19. (**E**) MTT assay of endothelial cells in response to H_2_O_2_ treatment with or without transfection of miR-19a-3p or AMO-19. (**F**) Immunofluorescence staining of ki67 in endothelial cells in response to H_2_O_2_ treatment with or without transfection of miR-19a-3p or AMO-19. (**G**) Western blot analysis of expression level of CD31 and HIF-1α in left ventricle of mice with or without MI. (**H**) Immunohistochemical staining of CD31 level in mice after MI. (**I**) TUNEL staining was performed to measure the level of endothelial cell death. (**J**) Immunofluorescence staining of ki67 in peri-infarct area of left ventricle. *P < 0.05 and **P < 0.01. All experiments were performed more than 4 biological repeats. Scar bar = 50μm.

Hypoxia inducible factor alpha (HIF-1α), a key cellular survival factor, plays crucial roles in response to hypoxia condition and has been identified to mediate important cardioprotective function. Furthermore, previous study has indicated that upregulated expression of HIF-1α promoted angiogenesis and resulted in increased tissue perfusion in animal models of ischemic cardiovascular disease [9, 10]. However, the relationship between miR-19a-3p and HIF-1α has not been indicated in endothelial cells. First, we used TargetScan (http://www.targetscan.org/) to forecast the potential interaction between miR-19a-3p and HIF-1α and one -binding site of miR-19a-3p in the 3’-UTR of HIF-1α mRNA was identified (Figure 4A). And as shown in Figure 4B, luciferase assay was performed and showed that miR-19a-3p, which had no effect on the vector that carries mutations of the binding site (HIF-1α-Mut), inhibited the activity of wild-type HIF-1α luciferase vector (HIF-1α-WT). To further identify HIF-1α as the target gene of miR-124 in endothelial cells, both loss-of-function and gain-of-function approaches were applied. AMO-19 increased the expression of HIF-1α in cultured endothelial cells, whereas miR-19a-3p alleviated the level of HIF-1α (Figure 4C). In contrast, co-treatment of miR-19a-3p and AMO-19 reversed the inhibition effect of miR-19a-3p transfection on the expression of HIF-1α (Figure 4C). These data suggest that HIF-1α may be one of the target genes of miR-19a-3p. Next, we explore if HIF-1α is involved in the attenuation of proliferation of endothelial cells induced by miR-19a-3p delivery. To confirm this, we transfected endothelial cells with either miR-19a-3p, AMO-19 or AMO-NC and then treated with H_2_O_2_. Western blot analysis showed that H2O2 increased HIF-1α protein levels, which was reversed by miR-19a-3p transfection (Figure 4D). Compared with NC group, AMO-19 significantly increased the expression level of HIF-1α (Figure 4D). MTT assay was performed to detect endothelial cell viability and it was inhibited in miR-19a-3p group. However, cell viability was elevated by administration of AMO-19 and interference of HIF-1α using siRNA significantly abolished this recovery effect (Figure 4E). Representative ki67 staining of endothelial cells with different treatment way were displayed in Figure 4F. Inhibition of HIF-1α expression attenuated the proliferation of endothelial cells caused by AMO-19 (Figure 4F). Then, *in vivo* study was also performed to investigate the role of miR-19a-3p on angiogenesis. Western blot showed that administration of antagomir-19 increased the protein levels of CD31 and HIF-1α, which was inhibited by AMI (Figure 4F). Co-infection of AAV5-shHIF-1α reversed the effect of AMO-19 and inhibited the level of CD31 and HIF-1α (Figure 4G). Moreover, immunohistochemical staining also indicated the elevation of CD31 level after antagomir-19 administration in mice with MI injury and reduction after knockdown of HIF-1α level (Figure 4H). Furthermore, TUNEL and the immunofluorescence staining results showed that administration of antagomir-19 inhibited cell death and promoted endothelial cell proliferation. And AAV5-shHIF-1α infection partially blocked the proliferation of endothelial cells and elevated the number of TUNEL positive cells compared with antagomiR-19a-3p administration group. (Figure 4I and 4J). These data show that downregulation of HIF-1α is involved in miR-19a-3p mediated inhibition of endothelial cells proliferation.

## DISCUSSION

Myocardial infarction is a common presentation for cardiovascular disease that leads to cardiac remodeling and eventually results in heart failure. Millions of people die from this kind of ischemic heart diseases. MicroRNAs are a kind of small noncoding RNAs that regulate gene expression by blocking translation or inducing degradation of mRNA. As it were reported for the first time in the 1990s, miRNAs research has quickly grown into one of the most famous area in biology. The role of miRNAs in MI has been widely determined. MiR-15 family members were the first to be reported that was upregulated after MI and induced death of cardiomyocytes [13]. In contrast, miR-24 was described as being a cardiac protective miRNA and forced expression of miR-24 reduced infarct size and improved heart function after MI [14, 15]. Our study indicated that expression level of miR-19a-3p was upregulated under ischemic condition. Besides, except acting on the cell itself, the role of miRNAs in cell-to-cell communication was also explored. miRNAs can be secreted and transmitted from cells to cells by multiple pathways and carriers and exosome is one of the most promising methods for clinical application among them. Previous study indicated that exosomes enriched in miR-126 and released from CD34^+^ cells promoted angiogenesis and improved heart function [16, 17]. However, the communication between cardiomyocytes and endothelial cells via the exosome containing miRNAs and secreted from cardiomyocytes is still poorly understood. In our work, cardiomyocytes released exosome was enriched with miR-19a-3p in response to 7 days MI and can be absorbed by endothelial cell. RT-PCR analysis showed that miR-19a-3p level in exosome addition group was higher than CTL and H2O2 treated group. These data suggested that the miR-19a-3p change induced by the injury of cardiomyocytes can be transferred into endothelial cells via exosomes.

Angiogenesis is a critical process in the tissue repair of MI. Promotion of angiogenesis is benefit for limiting infarct size and attenuating cell death. It was reported that inhibition of miR-92a resulted in significant growth of vessel [18]. Our study found that administration of those exosomes inhibited proliferation of endothelial cells and accelerated cell death, whereas transfection of AMO-19 reversed these effects. Our finding suggested that downregulation of miR-19a-3p potentially elevated endothelial cells proliferation and reduced cell death.

HIF-1α is an important transcription factor and play crucial role in the regulation of oxygen homeostasis [19]. HIF-1α regulates oxygen delivery and utilization via regulating vascular remodeling and angiogenesis, redox homeostasis and glucose metabolism. Previous study indicated that miR-223-3p attenuated the proliferation of ischemic endothelial cells and angiogenesis via inhibiting the expression of RPS6KB1 and the activation of downstream HIF-1α pathway [20]. Target prediction using TargetScan software showed that here is a binding site of miR-19a-3p in the 3’-UTR of HIF-1α mRNA. Here we reported that miR-19a-3p can inhibited the expression of HIF-1α and AMO-19 transfection abolished that effect. AMO-19 upregulated the protein level of CD31, which was suppressed in miR-19a-3p transfected endothelial cell. In contrast, the expression of CD31 was reduced in si-HIF-1α group. These data suggested that miR-19a-3p inhibited angiogenesis, at least in part, via targeting the expression of HIF-1α.

## CONCLUSIONS

In conclusion, the expression level of miR-19a-3p was increased after myocardial infarction. Exosomes including miR-19a-3p that secreted from cardiomyocytes can be absorbed by endothelial cell and inhibited the proliferation of endothelial cells via targeting the expression of HIF-1α, which was recovered by transfection of AMO-19. Moreover, Administration of antagomiR-19a-3p promoted angiogenesis and improved heart function in MI mice.

## MATERIALS AND METHODS

### Mice

The animal experiments were finished depended on the protocols. Male C57BL/6 mice were provided by Cyagen Biosciences (Suzhou, China) and divided into these groups randomly: Sham (control; sham-operated), AMI (MI model with LAD for 7 days), AMI +antagomir-19, AMI +antagomir-NC and AMI +antagomir-19+AAV5-shHIF-1α. MI model was established as follows [11]. Briefly, mice were anesthetized under intraperitoneal (i.p.) avertin and placed on a heating pad (37°C) in a supine position. Mice were intubated and ventilated with room air via a MiniVent Type 845 mouse ventilator (Hugo Sachs Elektronik-Harvard Apparatus, Germany). MI was established by permanent ligation of the left anterior descending artery (LAD) using a 7-0 prolene suture for 7 days. Sham group served as surgical controls and were subjected to the same procedures as MI mice with the exception that the LAD was not ligated. We used Antagomir-19 to inhibit expression of miR-19a-3p *in vivo*. We used a bulldog clamp to nip the aorta and then injected antagomir-19 into the left ventricle myocardium. In addition, Animals were treated with adeno-associated virus 5 carrying short hairpin RNA for HIF-1α (AAV5-shHIF-1α) (Biowit Technology, Shenzhen, China) via intratracheal injection in the dose of 1x10^11 vg per mice 7 days before MI.

All mice were raised in SPF animal houses. All the animals were intraperitoneally injected with 3% pentobarbital sodium and were killed by excessive anesthesia with a dose of 90 mL/kg. Left ventricle tissues were collected for biochemical assay. The experiments were finished depended on the protocols and accordance to the National Institutes of Health guidelines. This study was reviewed and approved by the Institutional animal care and use committee of Affiliated Hospital of North Sichuan Medical College.

### Cell culture

Primary neonatal mice cardiomyocytes (NMCMs)were isolated from newborn C57BL/6 mice heart by trypsin as follows. Newborn C57BL/6 mice hearts were cut off with scissors aseptically after the mice were disinfected in 75% alcohol and decapitated. Then the hearts were cut into small pieces in Dulbecco’s Modified Eagle Media (DMEM, HyClone, UT) without fetal bovine serum (FBS) and then were digested using 0.25% trypsin solution (Beyotime Biotechnology, Beijing). The digested cell suspensions were centrifuged at 1000 rpm, 5 min later we resuspended the cell deposition using DMEM medium containing 8% FBS. After 2 hours, we purified NMCMs via differential adhesion. Cardiomyocytes were cultured in cell incubator under a condition of 5% CO_2_ for 48 h.

### Transfection

MiR-19a-3p, negative control (NC) and Anti-miRNA oligonucleotide-19 (AMO-19) were produced by RiboBio (Guangzhou, China). Endothelial cells (HMEC-1) and NMCMs were transfected according to the protocol from manufacturer via X-treme GENE siRNA transfection reagent (Roche, Germany). Six hours later, we changed the transfection medium to culture medium with 10% FBS. After transfection for 48h, NMCMs and endothelial cells were treated with H_2_O_2_ (50 μM) for 3 h.

### Real time-PCR

Total RNA was isolated from serum and culture medium according to a standard protocol. And then, the purity and concentration of RNA was detected and all the samples were converted into cDNA using reverse transcription kit. We used SYBR Green (Thermo Fisher Scientific) system to perform the qRT-PCR. Data was analyzed by GraphPad 7.

### Western blot

Protein samples were blotted depended on standard protocol. And we used Odyssey Infrared Scanning System (Gene Co. Ltd., Hongkong, China) to scan the membranes. At last, we used Image J software to analyze the western bolt results.

The antibodies are as list: CD31 and GAPDH antibody were produced by Proteintech Group (Wuhan, China). HIF-1α antibody was produced by Cell Signaling Technology (Danvers, MA, USA). The secondary antibodies IRDye700/800 Mouse or Rabbit were produced by LICOR (Lincoln, Nebraska, USA).

### Luciferase reporter assay

psiCHECK-2 luciferase reporter plasmid was inserted with the wildtype HIF-1α-3’UTR or mutant HIF-1α-3’UTR sequences that contain the putative binding sites of miR-19a-3p. miR-CTL or miR-19a-3p mimics were transfected with reporter vectors into endothelial cells. The cells were collected after 48 h post-transfection and lysed to detect the luciferase activity (Promega).

### Echocardiograph

We anesthetized mice using 1% isoflurane-inhalation anesthesia. And then, we analyzed cardiac hemodynamics by suing a Vevo2100 system (VisualSonics, Toronto, Canada) 7 days after myocardial infarction. Mice were anesthetized and placed on a pad in a supine position and we used two-dimensional M-mode to assess left ventricular function and recorded using a 30MHZ transducer. Fractional shortening (FS) and ejection fraction (EF) were evaluated.

### Transmission electron microscopy

We purified exosomes from conditioned medium of NMCMs and depleted bovine exosomes of FBS by ultracentrifugation for at least 6 hours at 100,000 g. We collected the culture medium of NMCMs after 48 hours. Several centrifugations were performed to purify exosomes [12]. Briefly, we centrifuged the supernatant at 300 g for 10 minutes, 2,000 g for 10 minutes, and 10,000 g for 30 minutes. And then, we filtrated it through a 0.22-μm filter to eliminate cells, dead cells, and cellular debris. The supernatant was purified by ultracentrifugation at 100,000 g for 70 minutes and washed with PBS at 100,000 g for 70 minutes (Ultracentrifuge, Beckman Coulter, L8-70M). The exosome pellet was resuspended in 100 μl PBS for next experiments. NMCMs-derived exosomes were analyzed using exosome marker protein CD63 via flow cytometry and Western blot as previously described [12].

### TUNEL staining

We used the in-situ Cell Death Detection Kit (TUNEL fluorescence FITC kit, Roche, Germany) detect apoptotic. We used DAPI to stain nuclei. We used IX73 fluorescence microscope (Olympus, Valley, PA) to analyze fluorescence staining. We used Image-J to count the Total cells and TUNEL positive cells numbers.

### MTT assay

Endothelial cells were plated in 96-well plates and we used MTT assay to detect the cell viability. MTT (0.5 mg/mL; Beyotime Biotechnology, China) was added to every well after miRNA transfection and H_2_O_2_ treatment and incubated for 3 h at 37°C. And 150 μL DMSO was added and incubated for 15 min. We measured the absorbance by Spectrophotometer (Tecan, Austria) at 493 nm.

### Immunofluorescence staining

Endothelial cells were plated in a 24-well cell culture plate. After transfection of miRNA and H_2_O_2_ treatment, cells were washed by PBS and fixed with 4% paraformaldehyde. Cells were permeabilized with 0.2% Triton-X-100 solution in PBS. Next, we blocked cell using goat serum. Then, the cells were incubated with HIF-1α antibody at 4 °C overnight followed with FITC-conjugated goat anti-mouse antibodies incubation for 1h. After three washes with PBS, we incubated cells by DAPI.

Frozen sections of C57BL/6 mice heart were fixated in 4% paraformaldehyde and washed using PBS. We penetrated sections using 0.5% Triton X-100. After 3 times wash, we blocked sections with 50% goat serum. Then, sections were incubated with CD31 and ki67 antibody overnight. We incubated the sections using secondary antibody followed by DAPI staining.

Immunofluorescence was analyzed under an IX73 fluorescence microscope (Olympus, Valley, PA).

### Statistical analysis

All data is presented as a mean ± S.E.M. Statistical analysis was performed using Student's t-test or a one-way ANOVA.
